# Three-electrode galvanic microcells as a new antimicrobial tool

**DOI:** 10.1038/s41598-020-64410-9

**Published:** 2020-04-30

**Authors:** Wojciech Spisak, Andrzej Chlebicki, Mariusz Kaszczyszyn, Mateusz Szar, Jarosław Kozak, Arletta Olma

**Affiliations:** 1 Research & Development Centre ALCOR Ltd., Kępska 12, 45-130 Opole, Poland; 2 W. Szafer Institute of Botany, Polish Academy of Sciences, Lubicz 46, 31-512 Cracow, Poland

**Keywords:** Environmental biotechnology, Antifungal agents

## Abstract

This study presents the first research related to fungal and bacterial growth within electromagnetic fields generated by three-electrode galvanic cells, with PDA growth medium as an electrolyte. We used galvanic microcells constructed with copper, bismuth and zinc metal bars. The configuration of these electrodes was a fundamental agent in the creation of a maximum inhibition zone and in bismuth ion movement. Fungal strains, such as *Aspergillus tubingensis* and *Rhodotorula mucilaginosa*, and the bacterium *Micrococcus luteus* were used as model organisms.

## Introduction

It is necessary to ensure high hygienic standards in air-conditioned rooms and inside car cabins. Office buildings with air-conditioning systems consistently report sick building syndrome that is derived from microbial growth. Fungal and bacterial bioaerosols and mycotoxins can be the source of unpleasant odours and health problems for car drivers and passengers. A variety of safety considerations must be taken into account as part of any chemical application within an HVAC (heating, ventilation, air conditioning) system. Overuse of antimicrobial products can lead to unnecessary exposure to chemicals. A variety of studies have provided evidence indicating important connections between human health and the rapid emergence of multidrug-resistant pathogenic fungi^1^. Microbes present in an internal environment are permanently challenged by a number of chemical compounds. Antibiotic and biocide resistance is mainly caused by the colocalization and/or cotransfer of genes conferring multiple immunity mechanisms^2–4^. Mutations resulting in conformational changes to drug target sites are the most frequent form of resistance in harmful microorganisms such as fungi^5–7^. We should make every effort to stop this phenomena. Reduction in the use of biocidal substances is one of the possible solutions. To reduce our reliance on chemical control alone, we propose to use properties of galvanic microcells for the inhibition of microbial growth on hard surfaces within components of air handlers and duct interiors of HVAC systems. Biocontrol and biocidal activity of such arrangements will not be limited to chemical processes but will also include phenomena related to a generated electromagnetic field and forced movement of bioactive ion within the system.

The galvanic action between metals causes the oxidation of the anode metal (anodizing) to ions, which diffuse in the surrounding medium in the form of an ion stream. It is believed, but yet unsubstantiated, that galvanic action offers antimicrobial activity. To our knowledge, studies related to fungal growth within electromagnetic fields generated by galvanic cells with active metal electrodes are limited to two-electrode systems for fungal biocontrol by creating an ionic conductive environment which is generated electrochemically^8^. Galvanic systems are activated by excessive humidity and stay in a resting state under dry conditions. Zinc and copper are well-recognized antifungal metals with limited antibacterial activity. Another promising bioactive metal is bismuth. The medicinal application of bismuth and bismuth salts is focused in two fields: antimicrobial and anticancer agents^9^. Bismuth (III) complexes are often used in protection against bacteria. A number of bismuth salts working as a bismuth ion sources are used worldwide for medical antibacterial treatment and include colloidal bismuth subcitrate (CBS), bismuth subsalicylate (BSS), bismuth subnitrate and, more recently, rantidine bismuth citrate (RBC) (see^10^). Various bismuth salts have *in vitro* activity against *Helicobacter pylori*^10,11^.

Because of this previous research, we added a third bismuth electrode to a zinc and copper galvanic cell to investigate the possibility of using this three-metal electrode systems as a new galvanic microcell antimicrobial tool. We speculated that this arrangement of anodes and cathodes will enable partially increased movement of bismuth ions. Antimicrobial activity of the system was optimized for configurations and various distances between the electrodes of the used metals.

## Materials and Methods

### Electrodes for galvanic cells

Galvanic cells for experiments on the millimetre scale were constructed with electrodes in the form of 1.2-mm diam., 7-mm long bars fixed in the middle of polystyrene Petri dishes. Special high-grade zinc with a minimum zinc content of 99.5% was used for the anodes, and Electro Tough Pitch Copper with a minimum copper content of 99.5% was used for the cathodes^8^. Bismuth electrodes in the form of 1.2-mm diam. bars were cast from bismuth solid metal that was 99.7% pure. Electrodes were arranged in one line, within a 10-mm distance between the central axis of each of them.

### Finite element modelling (FEM) of the electric field

The FEM package used to create electric field models was Wolfram Mathematica R10 multiphysics (https://www.wolframalpha.com). The calculations were performed for standard electrode potential relative to the standard hydrogen electrode, with −0.76 V for zinc, +0.52 V for copper and +0.31 for bismuth. Spherically shaped electrodes with 1.2 mm diam. were used as simplest geometric models for electric field calculations.

### Fungi

Fungal strains from the genera *Aspergillus* (dematiaceous) and *Rhodotorula* (yeast) were used in experiments. These fungi are very often isolated from home and car air-conditioning systems. Standard Petri plates (90-mm diam.) containing PDA medium were used. After one week of incubation, fungal colonies were removed to fresh PDA medium and cultivated at room temperature under dark/light conditions. Our molecular analysis of the internal transcribed spacer (ITS1-5.8S-ITS2) identified the yeast as a *Rhodotorula mucilaginosa* strain with 98% identity to the sequence of *R. mucilaginosa*, GenBank accession number EU285542.1^8^. The *Aspergillus* section *Nigri* strain was identified after sequencing (ITS1-5.8S-ITS2) as *Aspergillus tubingensis* (Schöber) Mosseray (non-published data).

### Growth observations

Fungal mycelium with spores were transferred to 100-ml beakers containing 20 ml of 0.9% agar and stirred with a magnetic stirrer approximately every 30 sec. A solution with suspended conidia was sprayed on the medium surface (PDA medium, pH 5.6) in Petri plates containing metal electrodes using a pharmaceutical atomiser. The cultures were then maintained at room temperature in the dark. After five days, the diam. of the growing halo was measured. Plates were documented photographically using a Cyber-shot DSC-RX 100 camera. Observations were made after 3, 7, 10 and 20 days of growth. For microscopic observation and photography of fungal morphological structures, Nikon SMZ 1500 and Nikon Eclipse 80i light microscopes were used^8^.

### Bacteria

A bacterial strain of *Micrococcus luteus* (ATCC 4698) was used in the experiments. This gram-positive, obligate-aerobic and pigmented bacterium has ability to utilize succinic acid and process terpine-related compounds from natural amber as well as other hydrocarbons, among them petrol^12^. It is a very common bacterium isolated from human and mammalian skin, soil, dust, water and air^13^. It was also isolated from 120-million-year-old amber^14^. The yellow colour of colonies was very useful in our experiments, and degradation of hydrocarbons was an important characteristic of this strain.

### Inhibition zone

**Figure 1:**
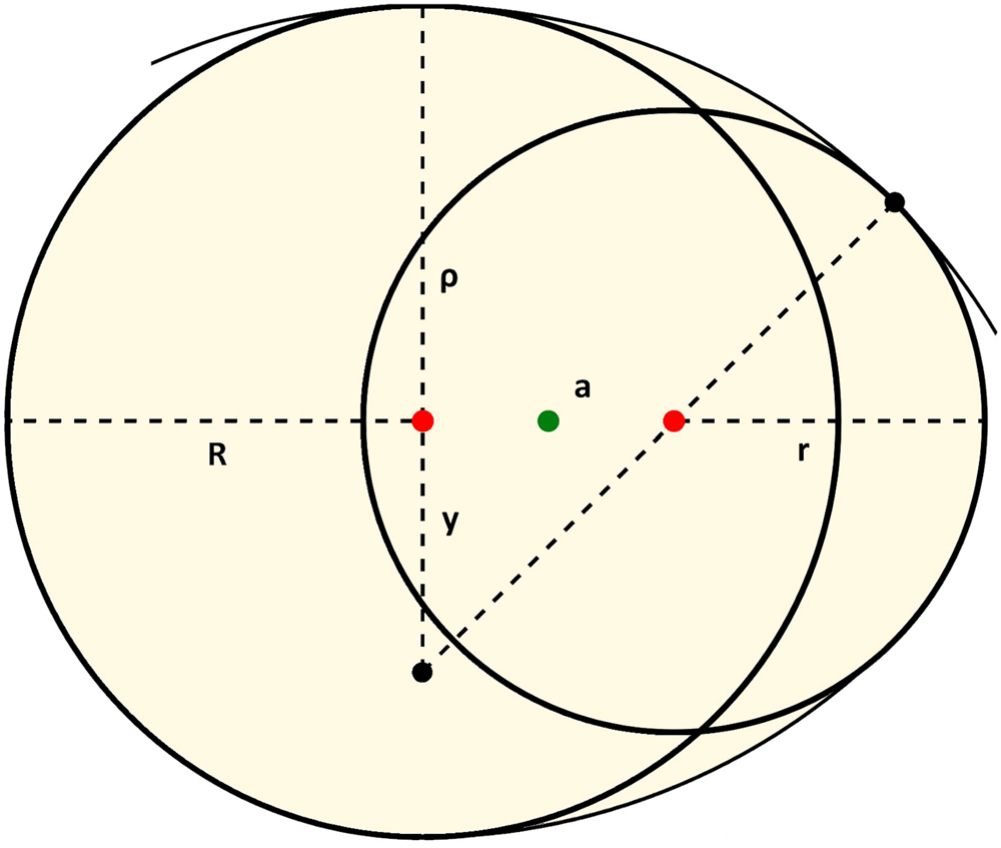
Geometry model of an oval^15^ used for calculation of inhibition zone areas.

Observed inhibition zones had oval shapes. The term oval when used to describe curves in geometry is not well defined. Many distinct curves are commonly called ovals when a plane curve resembles the outline of an egg or an ellipse. In our arrangement of the three-electrode system fixed in one line, the final ovals resulted from joining inhibition zones of particular electrodes, which form arcs of different radii. In this situation, an oval is constructed from two pairs of peripheral electrode arcs with two different radii (Fig. 1).

The arcs are joined at a point in which lines tangential to both joining arcs lie on the same line, thus making the shape smooth. For this geometry model, the area A (Fig. 1) enclosed by the oval can be calculated by formula

1 $$A=\frac{1}{2}\left[a(R-r)+\pi ({r}^{2}+{R}^{2})-\frac{{a}^{3}}{R-r}+\frac{[{a}^{2}+{(R-r)}^{2}]({a}^{2}-3{r}^{2}+2rR+{R}^{2})}{2{(R-r)}^{2}}{{\tan }}^{-1}\left(\frac{2a(R-r)}{{a}^{2}-{(R-r)}^{2}}\right)\right]$$

where **a** denotes the distance between peripheral electrodes and R and r denote the radii of inhibition zones created by peripheral electrodes (Fig. 1).

As expected, this formula reduces to the area of a circle^15^

2 $$A=\pi {R}^{2}\,for\,0\le a\le R-r$$

and to the area of a stadium

3 $$A=\pi {r}^{2}+2ar\,for\,r=R.$$

Bismuth concentration in medium

Analysis of the X-ray signals during SEM observation was used to map the distribution and estimate the abundance of bismuth in dry medium.

## Results and Discussion

Antibacterial and antifungal activities of the used three-electrode galvanic cells were evaluated based on the area of the inhibition zones determined by measured radii, R and r, according to used model of oval compass construction^16^. The shape and size of the inhibition zone depended on the used electrode configuration in the galvanic cells. Disc-shaped inhibition zones were observed only when bismuth was tested alone in a half-cell for determination of its oligodynamic propriety. We started the investigation of antimicrobial effects by using a half-cell, which is recognized in electrochemistry as a structure that contains a conductive electrode and a surrounding conductive medium (electrolyte) separated by a naturally occurring Helmholtz double-layer structure.

**Figure 2:**
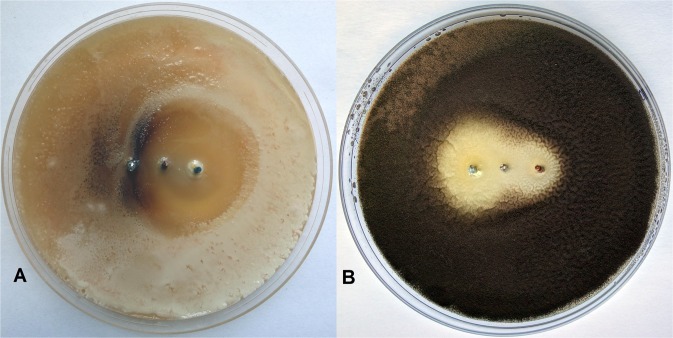
Inhibition zones in three-electrode galvanic cells for the bacterium *Micrococcus luteus* after 20 days (**A**) and the fungus *Aspergillus tubingensis* after 20 days (**B**).

Inhibition zones for the three-electrode galvanic cells were observed for both the bacterium *Micrococcus luteus* (Fig. 2A) and fungus *Aspergillus tubingensis* (Fig. 2B).

**Figure 3:**
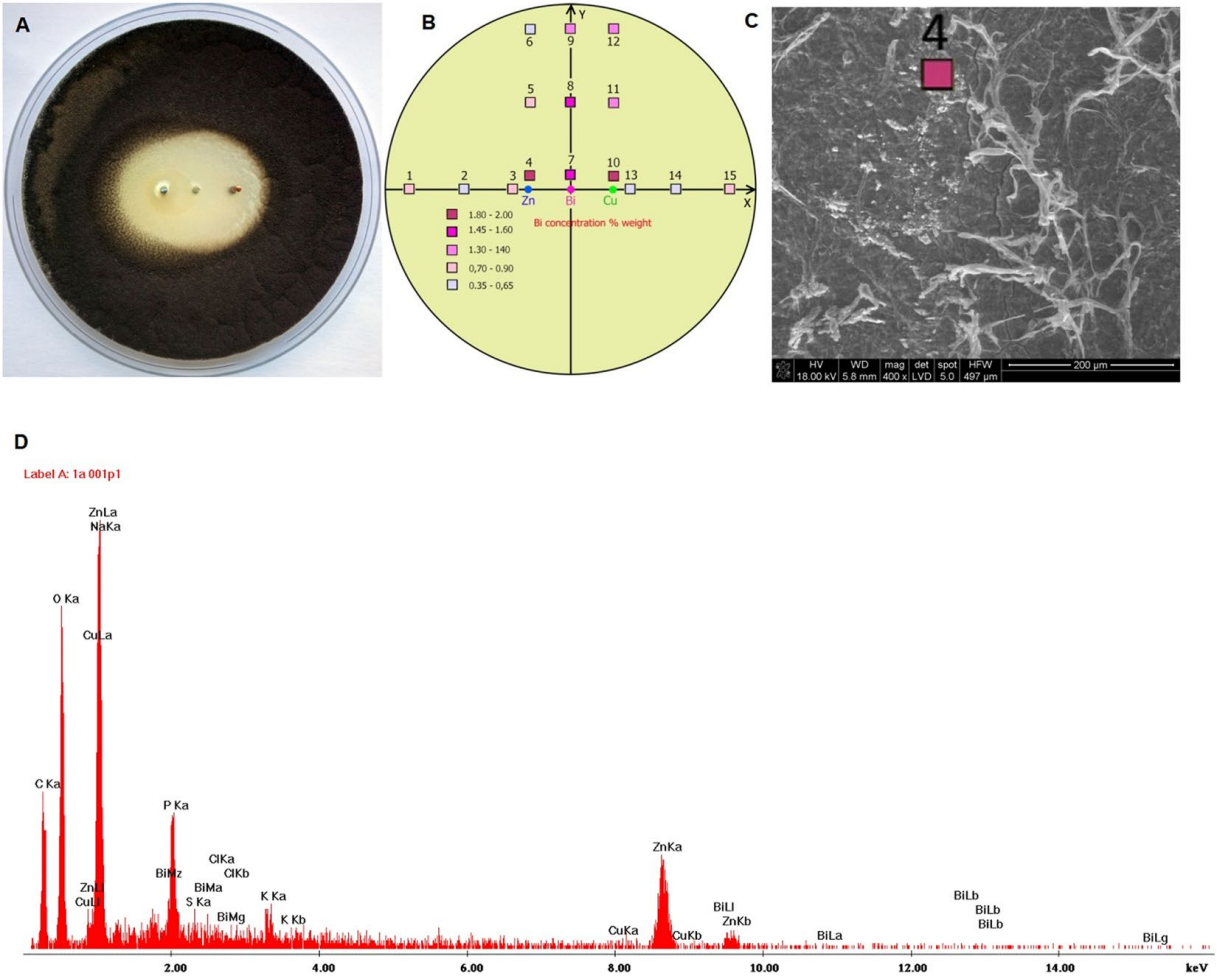
Observed inhibition zone generated by a zinc-bismuth-copper galvanic cell for *Aspergillus tubingensis* after 20 days of incubation (**A**) and the detected bismuth concentration (**B**). Microscopic view of the measurement point 4 (**C**) and the spectrum of detected elements in point 4 (**D**). Localization of measurement points (colour squares from Fig. 3B) in Petri dishes according to X-Y coordinates, with 0.0 as the bismuth electrode axis (see Table 1).

In the half-cell, bismuth ions were detected in the medium only in close proximity to the electrode, with a maximum concentration of 1.77% weight. At distances bigger than 5 mm, bismuth ions were not detected. When bismuth electrodes were fixed between zinc and copper electrodes, bismuth ions were detected in the entire medium (Fig. 3), with a concentration up to 1.96% weight.

**Table 1:** Localization of measurement points (coloured squares in Fig. 3D) and bismuth concentration in Petri dishes according to X-Y coordinates.

Point	X [mm]	Y [mm]	Bi [% weight]
1	−37	0	0.61
2	−25	0	0.49
3	−13	0	0,90
4	−10	3	1.96
5	−10	20	0.8
6	−10	37	0.35
7	0	3	1.51
8	0	20	1.49
9	0	37	1.18
10	10	3	1.8
11	10	20	1.34
12	10	37	1.4
13	13	0	0.48
14	25	0	0.48
15	37	0	0.74.

**Figure 4:**
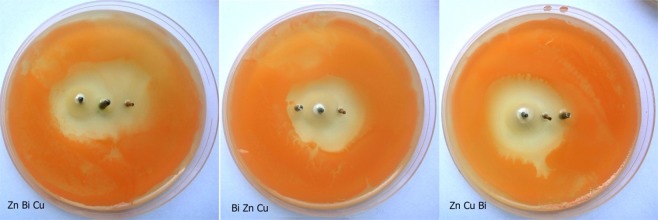
Inhibition zones of three-electrode combinations for *Rhodotorula mucilaginosa* after 10 days.

This observation confirmed the previous hypothesis that electromagnetic fields generated by galvanic cells may increase bismuth ion mobility in medium. In the next step, different arrangements of three electrodes, zinc-bismuth-copper, bismuth-zinc-copper and zinc-copper-bismuth, were tested (Fig. 4).

**Figure 5:**
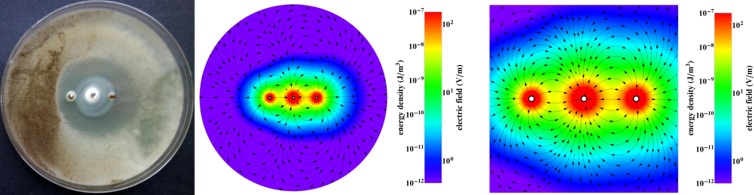
Inhibition zone for the electrode configuration Bi, Zn, Cu for *Aspergillus tubingensis* (after 7 days). Colours show the energy density and electric field.

**Figure 6:**
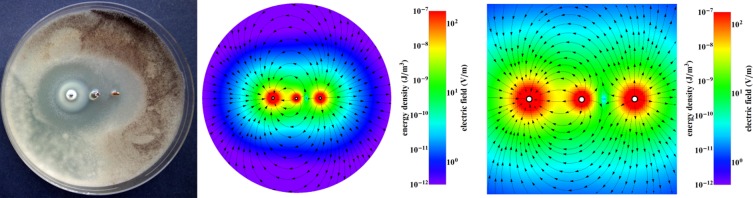
Inhibition zone for the electrode configuration Zn, Bi, Cu for *Aspergillus tubingensis* (after 7 days). Colours show the energy density and electric field.

**Figure 7:**
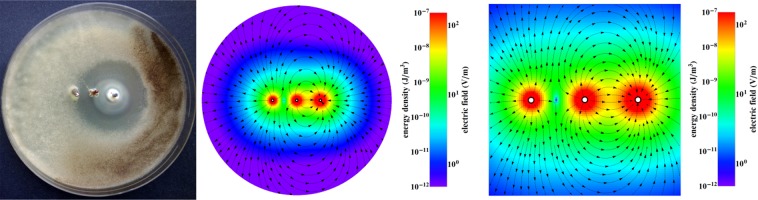
Inhibition zone for the electrode configuration Bi, Cu, Zn for *Aspergillus tubingensis* (after 7 days). Colours show the energy density and electric field.

We hypothesized that the unique geometric properties of the observed inhibition zones could be correlated with electromagnetic fields generated by the three-electrode galvanic cells and oligodynamic proprieties of the electrode metals. Some correlation could be observed (Figs. 5, 6 and 7). In the three-electrode system, we had a single zinc anode and two cathodes, bismuth and copper, according to zinc, but we considered that bismuth is an anode according to copper. The created electric field (Figs. 5, 6 and 7) assumed that Zn^2+^ ions are strongly mobile outside of the anode. Configuration of the electrodes has fundamental importance for obtaining a maximal inhibition zone (Figs. 5, 6 and 7).

**Figure 8:**
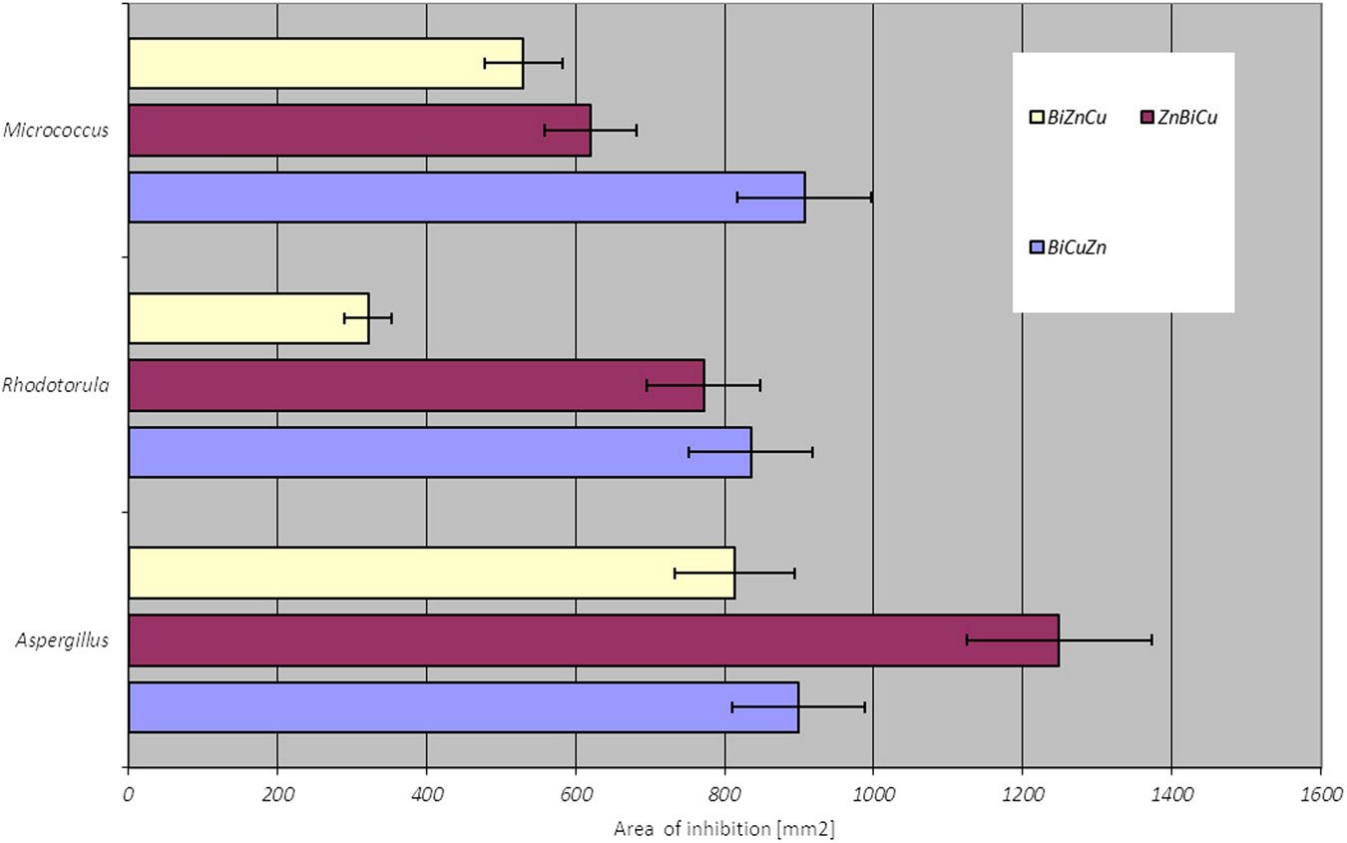
Area of inhibition zones according to electrode configuration.

Areas of observed inhibition zones were calculated according to oval model^15^ for each arrangement of electrodes (Fig. 8)

The arrangement of the electrodes and the addition of the third Bi electrode to the classic galvanic cell (Cu-Zn) enabled mobility of these ions (Fig. 3B) and increased inhibition of the growth of *Micrococcus luteus* (Fig. 2A). The energy density of electromagnetic fields generated by galvanic action is strongest between the zinc and copper electrodes. The localization of bismuth electrode between these two increase the bismuth ions mobility much more than in other layouts. Three electrodes galvanic systems are source of a bioactive “ ions cocktail”. The idea that the combination of well know antimicrobial zinc ions, with bioactive bismuth and copper ions, will result in a stronger antimicrobial effect is supported by world recommended three drugs “cocktails” antiretroviral therapy.

Our investigation has shown that electrode configurations have fundamental implications for obtaining an optimal inhibition zone. We also enhanced the mobility of bismuth ions, which is very important for inhibition of bacteria in biotechnological applications. Galvanic protection systems may have an application in the medical field as well, as it can be used in combination with conventional cleaning techniques in the reduction of biofilm formation on indoor hospital surfaces. Electrodes, in the form of micrometre size grains, fixed in a polymer matrix on protected surfaces seems to be promising in the prevention of infections acquired in hospitals and palliative care clinics
